# A grounded theory of female adolescents' dating experiences and factors influencing safety: the dynamics of the Circle

**DOI:** 10.1186/1472-6955-6-7

**Published:** 2007-09-20

**Authors:** Sharyl E Toscano

**Affiliations:** 1 Department of Nursing, College of Nursing and Health Sciences, University of Vermont, 106 Carrigan Dr., 212 Rowell, Burlington, VT, USA

## Abstract

**Background:**

This paper describes the nature and characteristics of the dating relationships of adolescent females, including any of their experiences of abuse.

**Methods:**

A grounded theory approach was used with 22 theoretically sampled female adolescents ages 15–18.

**Results:**

Several important themes emerged: Seven stages of dating consistently described the relationships of female adolescents. A circle consisting of two interacting same sex peer groups provided structure for each teen as they navigated the dating course. The circle was the central factor affecting a female adolescent's potential for risk or harm in dating relationships. Teens defined abuse as an act where the intention is to hurt. Having once succumbed to sexual pressure, teens felt unable to refuse sex in subsequent situations.

**Conclusion:**

An awareness of both the stages of dating and the dynamics of the circle will assist health care providers to plan and implement interventions in the female adolescent population. Study findings on factors and influences that support non-abusive versus abusive relationship might help identify female teens at risk and/or support interventions aimed at preventing dating violence.

## Background

According to Erikson, intimacy is achieved when the adolescent has developed the capacity to commit to a concrete affiliation and abide by the commitment, even if this means sacrifice and compromise [1]. Paul and White [2] describe three stages in the development of intimate relationships in late adolescence. These are: stage one, the self-focused stage in which the adolescent is concerned only with the relationship's effect on self; stage two, in which the focus becomes the role; and stage three, individuated connectedness. Elkind [3] described teens as becoming in love with love; their notion of love is idealistic and when the ideal doesn't match up to reality their early romantic encounters can be a shock. Ideally, accomplishment of these stages leads to healthy dating relationships. However, within this developmental process, adolescents might also experience negative and/or abusive relationships. The purpose of this study was to explore the nature and characteristics of adolescent females' dating relationships, including any experiences of abuse.

Much research on violence against women has been focused on the areas of marital violence, cohabitation, and violence in pregnancy. Research about dating couples has focused primarily on college age students with samples from colleges and universities. Interpersonal violence is occurring within adolescent dating relationships similar in form to that in adult relationships and constitutes a social problem worth investigation [4-11]. Differences in prevalence rates reported depend on the severity of the form of violence measured (physical versus verbal), age of sample, and means of collecting the information (interview, self-report, etc.). Findings from one study suggest an increased prevalence of dating violence in twelfth-grade compared to female adolescents in grades 9–12 [12].

Emotional and verbal abuse are reported more often than physical abuse in the adolescent population [9]. One study reported an increased incidence of actual physical violence compared to verbal threats of violence in adolescent relationships. Suggesting, physical violence is more prevalent than threatened violence and that there might not be a great amount of warning for physical violence occurring in the adolescent population [4]. Researchers have reported forms of physical violence in adolescent relationships that include behaviors such as punching, physical beatings, and threats with a deadly weapon [4,5]. Violence against teen girls caused considerable morbidity and mortality [13], including substance use, eating disorders, depression [11], early sexual behavior, pregnancy, and/or suicide [6]. Dating violence has also been associated with STD and HIV testing and diagnosis in adolescent girls [14].

Having multiple partners in an 18 month period [9], dating an older boy [4,15], and lack of academic affiliation [16] have all been associated with increased amounts of violence. Increased number of sexual intercourse partners [17], sexual favors, rejection, intoxication [4] or an association with peer-drinking [18], breakdown in conflict resolution [19], and jealousy [20] have also been suggested as both contributors to, and in one study justifications [21] of dating violence. Other reasons cited for the violence include anger, confusion, love, sadness, and, for a small percent (6%), hatred [22].

Data also suggest that teens most often fail to report dating violence [4,23,24] and approximately 50% report continuing in the relationship even after dating violence has occurred [11]. Female and/or younger adolescents are least likely to seek help in response to dating violence [25]. In a sample of public high school students, females experiencing severe dating violence reported suicide ideation or attempts, but didn't test lower on life-satisfaction measures [26]. This suggests that teens might view these acts and their response as a normal part of teen life.

Research suggests that teens may have difficulty identifying violence when it occurs within their own lives. Teens report knowing of a violent relationship much more than they report being in a violent relationship [23]. Teens may not define violent acts involving themselves as abuse or violence. Teens will often deny a violent relationship and then report in survey format numerous examples of events defined by the researchers as violent [8]. In one qualitative study, disagreement existed as to the definition of certain events as violent or non-violent [21].

Incidences of adolescent dating violence vary between studies, but all studies support the existence of this phenomenon within adolescent dating relationships similar in form to adult relationships. A concerning proportion that report violence also report staying in the violent relationship. However, evidence suggests that teens report knowing of a violent relationship more often than being in a violent relationship. This may reflect victims' denial or conflicting views of what constitutes violence in a dating relationship. Thus, it is necessary to study what teens define as abuse or violence in a dating relationship. Furthermore, it becomes necessary to include teens not involved in violent relationships in order to describe their views of this violence. Teens outside the violent relationship may have views different from those living with abusive situations.

## Methods

Straus and Corbin's [27] grounded theory approach was used to generate substantive theory about adolescent females' dating relationships. Grounded theory methods are based on symbolic interactionism. There exist three main premises or assumptions about symbolic interactionism. These are: humans act toward things based on the meanings they ascribe to those things: meaning given to a particular thing (event, person, act) is a result of social interaction; and meanings are created, evaluated, and modified via an interpretative process particular to the person and are used by individuals in dealing with their encounters. One particular event may occur in the lives of several people, but each person will ascribe different meaning to the event based on his/her own social interaction [28]. The paradigm of symbolic interactionism is presented here in order to provide an overlaying construct to support the use of grounded theory in the study of adolescent dating violence. Using this construct, intimacy and abuse are viewed as existing as a result of symbolic interactions within the context of the individual in society. By applying symbolic interactionism to the concept of adolescent dating, intimacy and abuse might exist as a result of symbolic interactions within the context of the individual in society. Both the perpetration and acceptance of abuse might exist in the adolescent dating relationship as a result of skewed perceptions in acquiring the knowledge and skills needed to develop and form healthy intimate relationships.

Grounded theory method generally begins with a question or area of study without any preformed concepts or connections. The method's aim is not only to generate theory, but also to generate theory that is grounded in the data. According to Strauss and Corbin [27], grounded theory is comprised of three main processes. These are description, conceptual ordering, and theorizing. During description, the researcher attempts to clarify an event or experience as perceived by the subject, in this case the adolescent. Conceptual ordering consists of organizing the data along common dimensions that become evident through data collection. Finally, theorizing consists of developing the concepts from the conceptual ordering process and relating them to each other in order to provide a theory that will explain or predict phenomena [27].

Twenty female adolescents ages 15 through 18 (age 18, n = 9; age 17, n = 4; age 16 n = 1; age 15 n = 6), attending one of two rural/suburban high schools located in Suffolk and Middlesex Counties in Massachusetts were selected. Older teens were deliberately selected because they had an increased ability to self reflect on their dating experiences. Ethnically, participants were homogenous (Native American N = 1/White N = 19). To be eligible for the study, adolescents were females who had at least one dating experience. Adolescent females who were married or parents were not eligible for inclusion in the study. The sampling procedures consisted of two phases as described by Strauss and Corbin [27]. In the open sampling phase, study participants were included in the study if they met the criteria and were willing to be interviewed. During this stage flyers were distributed for participant recruitment. In the second stage, relational sampling, participants were chosen based on concepts that emerged from analysis of earlier interviews. For example, in early interviews, teens that were dating older boys and/or dating for longer (greater than 6 months) reported negative dating experiences. In later interviews, teens dating older boys and or teens in relationships lasting more than six months were purposively sampled. Sample size was determined when theoretical saturation had been reached (no new themes or concepts emerged during the interview).

Data were collected through semi-structured interviews one hour in length. I personally conducted the interviews in a private setting during the students' study hall. These interviews were conducted with a four teen pilot group and guided by eight previously piloted questions to elicit information about each teen's dating experiences and observations of her friends dating experiences. These teens were introduced to the questions and their answers were evaluated for content. The content was analyzed as to whether following the interview guide led to data addressing the research aims. Use of the interview guide provided ample relevant data and pilot study participants commented on the clarity of the questions and suggested changes. One example from the interview guide was, "Tell me about you dating experiences: what is dating like for you? "; followed by, "How are your dating experiences similar or different from those of your friends?" The attributes these teens reported as constituting abuse were examined along with those influences the adolescent suggested foster or hinder abuse in dating relationships. In subsequent interviews, refinement of the initial guide also occurred in order to include new themes about adolescent dating as they emerged (Table 1).

**Table 1:** Initial interview guide

1. Tell me about your dating experiences: what is dating like for you? (What meaning does dating have, how does dating make you fell, how does it affect your behaviors, feeling, or emotions?)
2. How are your dating experiences similar or different from those of your friends? Tell me about their dating relationships.
3. What are the positive consequences of dating? What are the negative consequences of dating? Are these similar for other people your age?
4. Tell me about negative dating experiences you have had or negative experiences of your friends.
5. What constitutes abuse in a dating relationship, either your own or one that you know about? What occurs in response to the abuse?
6. What factors or influences do you feel contribute to a non-abusive dating relationship?
7. How do you relationships with family member influence your dating? How do their relationship with each other influence your dating?
8. How do your friends influence your dating?

The study proposal was reviewed and approved by the Boston College institutional review board. A letter of approval was obtained from school principals at both participating high schools. Confidentiality was maintained in both collection and reporting of the data (all names used in this article are fictitious). Written consent was obtained from each participating teen and her parent or legal guardian.

Data collection, analysis, and interpretation occurred concurrently using a grounded theory approach. Analysis adhered to the methods of Strauss and Corbin [27]. Field notes of observations, emerging categories, relationships or theories were recorded manually during interviews, followed by a review of the audiotape recordings and field notes before subsequent interviews. I clarified concepts formed from interview data and converged responses from participants. Data were transcribed and microanalysis was used to generate initial categories using open codes (Table 2). Axial coding consisted of relating these initial categories back to their subcategories (Table 3). Relational statements, grounded in the data, were connected using memos created during data analysis. Categories became saturated and concepts were fully defined. As categories emerged and evolved from the data, relationships between the categories were investigated via systematic comparison. Theoretical and operational notes directed the next step in the research process to finalize the theory [27].

**Table 2:** Examples of coding

**Code list**		**Numeric or thematic codes**
Age of dating partner	Intimacy fails to progress	Desire for intimacy
Altered peer opinion	Jealousy of peer group	Teens define what they will tolerate
Attributes of abuse	Jealousy within the couple	
Attributes of a confidant	Negative effects of dating	Staying in the relationship due to overwhelming desire for relationships based on preformed ideologies
Attributes of a positive relationship	Negative dating experience	
Contributing factors to abuse	Peer assessment	Hiding negative aspects of abuse
	Peer accepted norms	
	Peer rejected norms	
Contradiction	Peer response to abuse	
Contributes to negative experience	Affect of peer on dating	The group as a source of safety
	Positive dating experience	Clear definition of abuse – mismatched response to abuse
Contributes to positive experience	Physical abuse	
	Perceived threat	The "circle" thing source of protections
Dating experience	Positive breakup	
Desire for intimacy	Response to negative experience	Failure to progress (danger)
Definition of abuse		Sense of a priori knowledge
Definition of dating	Response to sexual pressures	Play fight versus abuse
Desired dating experience		Validation versus uncertainty
	Response to stocking	Searching for the prince
Development	Response to negative (subheadings below)	
Definition of intimacy		
Dating experience with older boy	Response to abuse	
	Attributes of confidant	
Developing intimacy	Response after knowing of abuse (not your own) physical, emotional, verbal, and sexual	
Dating configuration		
Dating differences		
Dating circle		
Emotional abuse	Staying in a negative relationship	
Experience of a confidant		
Family response to abuse	Sexual pressures	
Affect of family on dating	Stages of dating	
	School staff response to abuse	
Family response to a history of abuse		
Group dating roles		
Construct to follow appears		

**Table 3:** Axial coding sheet

**Phenomenon**	**Isolation**	**Uncertainty**	**Intimacy**
**Casual Conditions**	Uncertainty, Shame	Inability to apply definitions	Sharing of time, contact, and information
**Context**	Lack of connectedness to a circle	Distant from validation source (the circle)	Stages of dating, reciprocity
**Intervening conditions**	Congruence between self and circle, distance from circle	Congruence between self and circle; physical evidence	Circle norms, rules, and values
**Action/Interaction**	Circle or teen might attempt to connect with the circle	Validation via circle	Adherence to the dating stages as defined by the circle (see conditions)
**Consequences**	Continues to be isolated unless reconnected to the circle	Validation	Positive dating experience

**Phenomenon**	**The circle**	**Outside influences**	

**Casual Conditions**	2 interacting same sex peer groups	Adult connected to teen	
**Context**	Dating stages, group meeting, talking and exchanging, couple-group dating, dating outside the group, reintroduction to the group, breaking up, and reintroducing the self into the group Increasing amounts of shared time, contact, and information as the stages progress (see intimacy)	Shares previous connection to both the teen and the circle	
**Intervening conditions**	Circle norms, rules, and values congruent with teen, development and dating stage congruent with the circle	Assess and react to changes in dating; react to intervention attempts from the circle, influence environment	
**Action/Interaction**	Dating stages, validation	Circle accessed outside influence	
**Consequences**		Increased teen safety	

**Figure 1:**
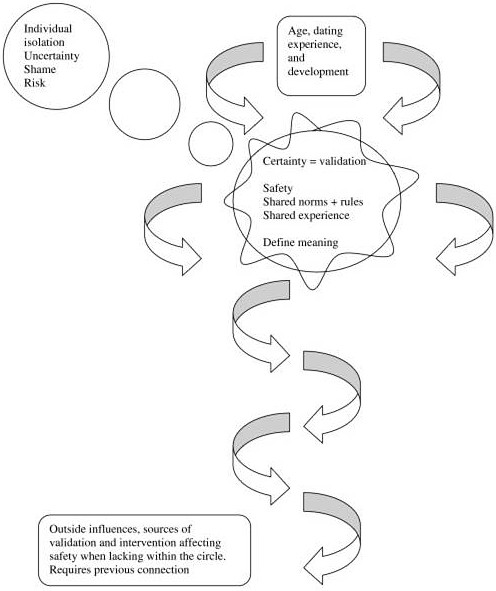
Theory of female adolescents' safety as determined by the dynamics of the Circle.

To ensure the credibility of the research process and findings all coded transcripts were sent to two researchers for review. Memos were created by grouping 2 or 3 coded interviews together. Memos included notes taken during the interviews. These memos were used for creating diagrams throughout the entire research process. Memos were dated, referenced to the data source they were derived from, and included concept headings. Memos evolved as the research progressed. All memos were collapsed by codes beginning to resemble each other during organization and sorting. Diagrams were created as relational statements between the concepts were formed via axial coding (Table 3) [27]. As categories became saturated and concepts were fully defined, relational statement connecting concepts emerged and a resultant theory emerged to explain these adolescent females' perceptions of the dating experience (Figure 1).

## Results

### Adolescents' perceptions of what constitutes a dating relationship

All of the reported results were derived inductively from the interviews. These female adolescent participants overwhelmingly agreed on seven stages of dating: group meeting, talking and exchanging, couple-group dating, dating outside the group, re-entering the group as a couple, breaking up, and reintroducing the self into the group.

### Dating stages

Stage one, group meeting, existed when one same-sex peer group began to interact socially with an opposite sex same-sex peer group. These same-sex interacting peer groups constitute the Circle. The majority of participants reported meeting and dating exclusively within these Circles. In the second stage, talking and exchanging, the two got acquainted with each other outside the Circle. Most often, this consisted of talking on the phone and/or chatting on the Internet. During these exchanges the two shared time, contact, and information independent of the Circle. When successful, the couple advanced to the third stage of dating (couple-group dating). In the couple-group dating stage, couples went on more formal dates with other couples within their Circle. Physical contact was limited. In stage four, dating outside the group, the couple went out independent of the group. During this time, they experienced an increased amount of closeness (physical or emotional), and an increased amount of shared time, contact, and information as compared to stage two. In stage five, re-entering the group as a couple, the two were reintroduced into the Circle as a couple. They maintained their independent relationship, but continue to maintain relationships within the Circle. This added a new role for both members of the couple. They were both individual members of the Circle and couple members. This commonly occurred concurrently, as the couple decided to label themselves as boyfriend/girlfriend. Teen participants described this as a time of discomfort, as they tried to achieve balance between their role as a couple and their roles within the group. Once this adaptation to new roles took place, the couple achieved the full support of the Circle.

Stage six was breaking up. Participants reported that, in a successful break up, the couple usually decreased their amount of shared time, contact, and information. The Circle was often involved in the break up process. In the final stage, reintroducing the self into the group, the two members of the couple became independent members of the group only. This involved a change in roles. It was normal for the former couple to continue some independent interaction, but they also began talking and exchanging with other group members. This shared contact continued as long as hope for reunion existed. Commonly, the two remained friends and returned to dating within the Circle. This was an important stage for the Circle's survival. Teen participants moved through dating stages quickly and they tended to have many short-term relationships.

Although the teen participants agreed on the specific stages, their opinions varied as to the seriousness of each stage, the specific intimate activities of the stage, and the exclusivity rule (dating only one person). These differences were determined by the norms and rules of the Circle. For example, some groups reported sex parties or sexual experimentation without emotional ties or commitments occurring during stage one and two [29], whereas other groups became physical sexually only after stage four or never. The time spent in each stage, the level of information shared, and/or the degree of intimacy experienced during each stage depended on the previous dating experience of both teens, as well as developmental age. Therefore, while there was overwhelming consistent agreement on the actual dating stages and their progression, the activities engaged in (intimate touch versus sexual penetration) adhered strongly to group norms which were determined by each particular Circle.

### Developing intimacy

Female adolescent participants desired to have a dating relationship. This desire was so overwhelming that in the early stages, teens were not choosy about the specific attributes of their potential dating partners. Having a boyfriend was seen as leading to increased popularity, self-esteem, and ability to experiment physically. For example, Alley shared, "When you're younger, all the popular girls have boyfriends and you want to have a boyfriend too because you want to be a popular girl." Participants' early relationships consisted of little or no emotional connection. Young teens without prior dating experience enjoyed dating even when they didn't like the boys they were dating. Second to having a boyfriend, these teens coveted long-term relationships.

Teen participants placed an extremely high premium on the length of their relationships. Missy believed that, " . . . if you have a long relationship you get kind of attached and it's more than just like a friend thing." In early stages of dating, particular attributes of the relationship were less important than having that relationship. Later, teen participants became more selective. This usually coincided with the acquisition of dating experience and a change in the teens' social development. Older teens' desire for intimacy superseded their desire to date. Lack of progression in emotional and/or physical intimacy led to the dissolution of the relationship. The important factor contributing to the formation of intimacy was spending time away from the group. In this study, the expectation for the development of intimacy coincided with the naming of the relationship. Even teens dating many boys were ultimately looking for intimacy. They emphasized that they would date only one boy if they found a connection. They put particular emphasis on the reciprocity of this connectedness. Alley said, "I'm in awe of him and I'm like, 'Oh ya you're home.' But then he comes in and we just watch movies and I'm like, 'Wow, I wish I could talk to you.' " Physical intimacy was not seen as a way to obtain emotional intimacy. However, they admitted that teens would become physical in an attempt to connect emotionally, but reported that this tactic usually failed. Erin said this about intimacy: " [intimacy is] just having a connection with each other." To be considered serious, it seemed there must be some form of physical intimacy. Rachael said this, "I think when it's physical alone, that can be pretty serious, but physical and emotional kind of like to me is what a serious relationship is . . ."

### What constitutes abuse in a dating relationship?

Teen participants' definitions of abuse varied; however, most teen participants agreed that in abuse, the intention was to hurt. This included verbal, physical, and emotional hurting. Teens most often used the term verbal abuse when talking about specific things that a person said. Emotional abuse was not always verbal, but included verbal cues. Emotional abuse required elements of consistent abuse over time. Control was presented as the most important factor mediating emotional abuse. Teen participants' overwhelming desire to have relationships made them more susceptible to control. Their partners used isolation as a means to gain control. Isolation from the Circle was an important way to gain control in abusive relationships, as it prevented the victim from leaving the relationship, blocked interventions from the Circle, and prevented information about the abusive relationship from passing from a Circle member to a victimized teens' parent(s). Once intimacy was established, the abuser was able to further control the teen by making suicidal threats. This type of emotional abuse was used to keep the teen from ending the relationship and was often used over the phone or after break ups, when a physical threat was not possible. Trisha stated, " . . . he didn't have to say it at all. I knew what he was thinking, like, 'You better watch out', or like, 'You might not see me here next week.' " Teens stated that emotional abuse was the most common form of abuse existing in high school.

Physical abuse within this study included hitting, touching in harmful ways, and physical threats. Teen participants had clear definitions of physical abuse; their convictions wavered if they became uncertain about whether the abuser intended to hurt. Intention was the key factor necessary to define something as abuse. This was true of emotional and verbal abuse as well. The teen's level of uncertainty as to whether the abuser intended to hurt her, dictated how she would respond to abuse.

Sexual abuse was difficult for teen participants to define. All of the teen participants talked about sexual pressures existing to varying degrees in their relationships. The presence or amount of sexual pressure experienced was associated with a lack of communication, a lack of or limited degree of parental structure, and the experience of dating an older boy. Once teen participants succumbed to sexual pressure, they felt they didn't have the right to refuse the second time. If they were coerced into sex the first time, they were expected to participate freely from then on. The main factor involved in succumbing to sexual pressures was the fear that if they didn't, they would lose the relationship. Diane said this about staying in an abusive relationship after having sex, "When you finally open up to someone and you get so close to them it just doesn't matter if they treat you like crap."

### Factors and influences that foster non-abusive relationships

Teen participants in the study described positive relationships as existing when couples shared common beliefs, values, and interests. Positive relationships were distinctive from friendships in the amount of emotional investment, time and the level of connectedness between the couple. A positive relationship was one that lasted a long time, consisted of emotional and physical closeness, included mutual respect and good communication, was comfortable, and fun. A positive date was someone who was a friend, believed in the teen participant, was honest, trustworthy, shared mutual interest with the teen, and was attractive, funny, happy, unique, considerate, and helpful. Other attributes that contributed to a positive relationship included: fostering the teen's ability to be herself, improving her self-esteem, and improving her self-confidence. Also, teen participants expected better results if their circle of friends liked their dates.

### Factors and influences that foster abusive relationships

Abuse occurred at an accelerated rate of frequency during times of increased stress in participants' relationships. This was usually at the beginning and at the end of their relationships. A smaller increase in risk existed at the transitional role stage, as the couple re-entered the Circle as couple members. If the boyfriend had not been a member of the Circle and the Circle was unwilling to accept him as a member, the teen had to decide whether to continue the relationship with her boyfriend or with the Circle. If she chose the boyfriend, she would become isolated from the Circle. Isolation from or lack of membership in a Circle contributed to the development of abusive behaviors by enabling negative events. Once in an abusive relationship, the teen was further controlled due to a lack of affiliation with the Circle and the protection provided by the Circle. Teens became isolated from parents, as well, due to shame. Teens reported hiding the abuse. Natalie said this about her friend's abuse, " . . . he would call her names and stuff first and when he was alone obviously he hit her because then she would come to school with bruises . . . she didn't want to tell me." Trisha stated, " . . . nobody knew exactly what was going on . . . I couldn't share that with anybody . . . it's so embarrassing . . . they very much hated him, um I think at times they hated me for being with him."

Negative experiences included: a feeling of being controlled, a possessive boyfriend, a jealous boyfriend, a boyfriend who was not incorporated into the Circle, fighting (verbal, physical), dependency on the boyfriend, a boyfriend who was an angry person, an untruthful and/or unfaithful boyfriend, a couple who were jealous of each other, an abusive date, and/or a date who had a violent history. Teens believed alcohol, family problems, and/or anger problems contributed to relationships becoming negative. Teens overwhelmingly agreed that the victim herself contributed by not knowing the abuser's limits. She was suspected of pushing his limits until he lost control. Also, she was seen as being desperate for not leaving. Uncertainty contributed to the lack of clarity when defining negative relationships. For example, teens consider jealousy to be both a positive and a negative attribute of dating. The correct amount of jealousy was equal to love, whereas too much was seen as controlling and negative if not abusive. Kelley said this about a relationship without jealousy, "That would be pretty boring." Also, controlling behaviors were interpreted as protection. Protection was seen as a positive dating attribute

For many participant adolescents, their relationships became negative and/or abusive only during the break up stage. Rachael said, "When I tried to break up with him, he would call me in the middle of the night drunk, telling me that he wanted me to die." A connection to their Circle was extremely important when breaking up. Negative and/or abusive experiences were more common when the teen lacked affiliation to a Circle. In order to succeed at breaking up the female teen must: accept that she can't fix her date; end any continued contact with her date; accept the loss of the ideal relationship; and re-affiliate or become affiliated with a peer group. Continued contact following a break up in a negative and/or abusive relationship was used as a way to maintain control. Dating an older boy increased teens' risks of being involved in negative and/or abusive relationships. This resulted from the isolating effect of dating an older boy, particularly where the boy was not a member of the Circle and did not enter the Circle after forming the relationship. This risk was similar to the risk of dating a boy from outside the Circle; however, it was different in that dating an older boy also posed other specific risks. Older boys experienced increased amounts of freedom; the teens report not having been ready for this. Older boys had increased sexual expectations that often led to the teen feeling increased amounts of pressure to become sexually active. Angela said this about having sex with an older boy, "And sometimes they expect more . . . and if you really want to be with this kid, you know he's good looking and he can have any girl . . . it would be awesome to still date you so you just do things. "

### Reactions to negative dating experiences and/or abuse

Participants' reactions to both negative and abusive dating experiences depended on the amount of uncertainty they felt about the negative and/or abusive event. When teens were uncertain they would seek validation from a secondary source. The teens needed to have physical signs present, thereby decreasing their level of uncertainty. Only then, would they seek validation from a secondary source. As a result, teens only responded to absolute cases that involved physical signs of harm; they were least likely to act or intervene in cases of emotional or verbal abuse where physical signs were absent.

In order to meet the critical certainty level, the intention to hurt must exist. Play fighting, for example, existed as a normal part of participant adolescent relationships. Play fighting was normally a playful act aimed at achieving closeness when the teen was not developmentally ready for intimate physical contact. This was very common in younger teen participants without much dating experience or those in new relationships. Participant teens viewed play fighting as mostly positive. Uncertainty existed when a teen became hurt in a play fight. Monica talked about her experience with play fighting, "Like I tell him to stop but he doesn't . . . [it's] just a joke and then it will end up being serious. It's not like out of anger or anything it's just like playing and it gets too hard . . . he doesn't mean to hurt, but he doesn't know his own strength." When there was uncertainty as to whether the boyfriend meant to cause harm; the young women relied on a priori knowledge.

Participant teens described a priori knowledge they had about their relationships and the relationships of their friends. This a priori sense, when relied on, acted to increase the level of uncertainty experienced by the teen, who herself was experiencing an abusive or negative dating experience. Teens who became isolated from the group exhibited an increased reliance on a priori knowledge, thereby increasing their risk of experiencing abuse and/or negative experiences. Teens' reliance on a priori knowing placed blame on the victim for not knowing beforehand that her boyfriend would become abusive. Natalie stated, "I'd avoid violent relationships. I'd never get into one of those . . . you know that a person gets angry really easily . . . don't say anything, because if you do then obviously it's kind of your fault."

Once the certainty level was reached, the Circle was the main source of validation for teen participants. In some cases, the group was actively involved in the dissolution of the relationship. This was highly dependent on the connection the teen had with the Circle. If she had become isolated from the Circle, the Circle might not act. In cases where an increased threat of danger existed, the Circle might act by accessing a parent or guardian. If the Circle acted in this way, the danger was very high, because their certainty level must be very high to risk not being validated. In these cases, teens expected a parent to act on their behalf. If parents failed to act, the Circle would feel that their claims were not validated and further attempts at intervention might not take place. A prerequisite to accessing a parent or guardian was having had a previous relationship with that parent or guardian. Teens would not access parents that they didn't know. This was true when teens accessed other adults as well, such as school nurses, guidance, or other school officials.

### Adolescents' reaction to negative and/or abusive dating experiences

Participant teens placed a great amount of importance on the length of time a relationship lasted. Because of this, teens reported being extremely reluctant to leave long-term relationships even if they were negative or abusive. These teens were more likely to stay in a negative and/or abusive relationship if the relationship was sexual. Teens reported a fear of getting a reputation and/or a fear that the boy might tell other boys about their performance. This seemed to be more important for younger teens. Teens also reported staying in long-term negative and/or abusive relationships because they were uncertain about their expectations of a relationship. It often was their first experience and they didn't have a good model for how the relationship might work better. Teens overwhelmingly desired the relationship, which made leaving difficult. They had invested time and they usually felt they could fix a negative or abusive relationship. Monica said, "He gets violent, but he's not as bad, he's gotten so much better."

Participant teens generally admitted to adhering to the first time rule (forgiveness of first abusive event) even in the hypothetical. The first time rule existed as a measure to protect the relationship and depended on teens' perceptions that they can fix negative and/or abusive relationships. Some justification for the abuse was required for application of the first time rule, however, tenuous. Angel said this, " . . . the first time I'd be like taken by surprise, obviously, but if I really had strong feelings for him I think I'd let it slide." Losses of control were acceptable reasons for violence and attributes of the girlfriend were often used to explain or defend losses of control. In this study, teens with a previous personal history of dating abuse were the only exception to acceptance of the first time rule. Jennifer said, ". . . once you've been abused it's kind of . . . it scars you and it's really like you're afraid . . . just one hit can change everything"

### Theory of female adolescents' safety as determined by the dynamics of the Circle (Figure 1)

This grounded theory is based on themes emerging from the study data. Four major concepts were developed in theory construction. These include: the circle of friends, individual characteristics, the isolated teen at risk, and outside influences. This theory is meant to explain the factors associated with actual and potential safety for female adolescents in dating relationships.

The Circle is the central factor affecting female adolescents' potential for risk or harm in dating relationships. The Circle, consisting of two interacting same sex peer groups, provides structure as the teen navigates the dating course. The Circle provides experience and is experiencing and creating meaning for the teen over time. Norms and rules are created within the Circle and are shared by the members of the Circle, leading to an increased level of safety from those not sharing these norms, rules, and values. The Circle validates the teen, thereby decreasing her level of uncertainty. Each member's experience depends on age, dating experience, and development, as these interact with the Circle over time. In the model, the arrows represent circular movement forward through time. With the passing of time, each member and the circle as a whole change and cannot return to a previous state. Any time the individual age, dating experience, or development is not in sync with the norms and rules of the Circle, the teen is at risk of becoming isolated from her Circle. This might also result from a violation or lack of adherence to Circle rules and/or norms.

Once isolated, uncertainty is the main factor affecting the female adolescents' potential for risk or harm in a dating relationship. Uncertainty creates an environment wherein the teen is unable to act in harmful and/or abusive relationships. Isolation from the Circle increases uncertainty, thereby increasing risk. Time reinforces isolation from the Circle, resulting in further isolation of the teen. Certainty will take more time to attain when isolated from the Circle. Once achieved, the teen has spent so much time away from her Circle that she feels shame. Increased time, shame, and uncertainty act to further isolate the teen. In the model, the increasing size of the circles represents growing risk over time and emotional distance.

A teen may also experience an increased risk as she separates from the Circle while dating independently with another member of the group. This is depicted in the model by a curved line entering and leaving the circle at intervals. Her close connection to the group protects her from most of this risk as long as she feels certain enough to seek validation from other Circle members. This certainty increases when physical signs of harm are present or imminent danger is perceived. Because of this, the Circle may act to hide less obvious forms of abuse; the teen lacks the level of certainty needed to access another member of the group, so she hides the experience from the group.

Norms and rules of the Circle prevent and deal with potential or actual negative and/or abusive relationships. If unsuccessful, the Circle will access an adult. This will only happen in the cases where the Circle perceives severe harm. If the adult chooses not to act, he or she will cut off connection to the Circle and attempts at intervention will stop. The Circle will only access an adult that they have had a previous relationship with, one with whom they have connected by sharing personal information. This relationship is depicted in the model by a helix leading toward adults. The helix denotes that the relationship is accessed one way, teen to adult in times of danger. The circular motion represents movement across time and, therefore, a change in the relationship over time.

## Discussion

The basic premises of symbolic interactionism are that: humans act towards things based on the meanings they ascribe those things; meaning given to a particular thing is a result of social interactions; and meanings are created, evaluated, and modified via an interpretative process particular to the person [28]. The study findings support a view that the meaning ascribed to an individual's dating process results from the balance between the individual freedom of the teen and the social norms and rules required by membership in her Circle. Seemingly, greater social structures, including school, family, and community groups contribute meaning within the dating process. However, the current study findings clearly support the small role of these contributions in comparison to the enormous effect of the Circle. Meanings are constructed, modified, and persist as a consequence of membership or lack of membership in the Circle and are formulated based on the experience of being a member of that Circle. This is evident as teens define and respond to dating experiences based on the meaning constructed via their interactions as members of the Circle. Awareness of meaning within the social context of a Circle explains the variation of action and reaction to varying levels of safety in dating relationships. Dating has a different meaning for each teen; this meaning is created and influenced by her membership in a Circle, and her action and reaction are supported or rejected by that same Circle. The consequences of her actions and reactions result in negotiations between her and the Circle that act to further define and construct meaning of the dating process for the teen.

There is another model in the relevant literature that aims to explain the cycle of adolescent violence. That model, entitled the cycle of adolescent violence, was created using concepts existing in the literature [30]. The model suggests that within a dating relationship, both persons bring to that relationship certain genetic and personality factors and environmental and socialization factors. The dating relationship is also affected by dating history and self-perceptions. All these factors interact to respond to a conflict in the dating relationship. Situational attributes (length of relationship, drug use, and/or stress), cognitive attributes (attitudes or beliefs about violence, shame), and psychological attributes (coping skills and self-esteem) all contribute to whether the conflict will have a violent or a non-violent outcome. Both outcomes result in staying in the relationship or ending the relationship. If the teen chooses to stay in the relationship, then this experience feeds back into and becomes connected to the next conflict. If the teen chooses to leave the relationship, then the experience feeds back and become connected to the next dating relationship [30]. Many of the components of that model and the resultant theory of this study are similar. However, the cycle of adolescent dating violence model suggests that a conflict occurs followed by the decision to stay together or break off the relationship whereas the resultant theory of this study suggest that the break up itself might be the conflict that initiates the violence. Furthermore, findings from this study suggest that the circle is central to this process, whereas the cycle of adolescent dating violence places the circle role socialization of seemingly equal value with family and community socialization. Both represent important contributions to the study of adolescent dating relationships. Differences may exist as a result of data gathered from an external perspective (that of the researcher) and data gathered from an inward perspective (that of the adolescent teen). Furthermore, this study is inductively derived whereas the cycle of adolescent dating violence was derived using deductively deduced existing knowledge.

One qualitative study in the relevant literature, Lavoie et al [21], interviewed teens within their peer group when possible in an attempt to extract the group experience of dating. Their discussions included the topics of love, adolescent heterosexual couple relationships, and violence within these relationships [21]. Consistent with the methods of this study, those researchers encouraged discussion of relationship that members of their sample knew about. Several of their findings are consistent with the findings of this study. Disagreement existed as to defining certain events as violent or non-violent. This may result, as it did in this study, from a struggle with applying definitions to actual situations. Lavoie et al [21] also found that violence was more acceptable if the abuser had been frustrated and/or lost control. Abuse was justified if some reason existed for this loss of control. Examples included jealousy and use of alcohol. Also, attributes of the girl seemed to lead to violent situations; one of these attributes was a strong need for affiliation [21]. In this study, this was represented as an overwhelming desire for the relationship. Similarly, subjects in the Lavoie et al [21] study discussed the threats of separation and reprisals including breaking off the relationship. In this study, teens expressed a general fear of losing the relationship acting to keep teens in negative and/or abusive relationships. Similarly to the subjects in this study, their subjects suggested that communication problems might result in violence and that the victim might provoke this reaction of violence. Most important is the finding in the study by Lavoie et al. that a teen would remain in a relationship due to her affiliation needs with the group and fear that breaking up with the boy would compromise her position in the group [21]. This became the main focus from this study data as the circle became the central concept mitigating safety for adolescent females in dating relationships.

One finding from Lavoie et al [21] study, that violent boys come from violent groups, did not emerge in this study data. However, the concept of dating rules and norms being formed within the circle would suggest the formation of violent circles is possible. There is additional support in other relevant research findings that report the presence of friend violence as a predictor of later dating violence [31]. The data from that study suggested that rough sex exists as a consensual form of violence. This may exist at the other end of the spectrum from the play fighting that emerged from this data.

A qualitative study using a feminist perspective supports and may further explain some of the research findings. The data from Berman et al. suggested that girls' tolerance of sexual harassment results from a desire to maintain relationships with their boyfriends [32]. Findings from this study suggest that teens' desire to maintain the relationship is responsible for tolerance of most forms of negative and/or abusive relationships. Also, results from this research reflect the overwhelming importance for adults to act if teens access them for help. The Berman et al. study also found that adults failed to act and instead punished the girls for reacting [32]. In addition, the Berman et al. study sample reported references to sexual harassment occurring in the data, but were instead labeled as teasing by study participants [32]. Similarly, data findings in this present study suggest that teens have difficulty applying any negative and/or abusive definitions to their own experience.

The present study was designed in a manner that would reflect the average dating experience. A similar study including only females with a positive history of dating violence may contribute to knowledge of violent or abusive dating relationships. Including male teen participants in future studies might further explicate their perceptions of roles, rules and norms within the circle. This model also needs additional testing in diverse ethnic groups. Researchers might foster the development of knowledge about adolescent dating violence by including inductively derived concepts from qualitative research in their research design.

## Conclusion

This qualitative research study has advanced the knowledge of adolescent dating by delineating the dating stages, specifying the dynamics of group interaction related to dating, and providing definitions of abuse and negative dating relationships as perceived by the participating teens. The study generated knowledge about adolescent women's perceptions of factors and influences that foster non-abusive relationships and those factors and influences supporting abusive relationships. These teens described the actions they have taken in response to negative dating experiences. The interview itself seemed to have the effect of shaping and expanding teens' definitions of negative and/or abusive relationships. Any interventions should include this sort of self and group reflection. Interventions that fail to consider or include the dynamics of the Circle would probably be unsuccessful. A grounded theory emerged to explain the characteristics supporting safety versus risk in high school dating relationships.

## Competing interests

The author(s) declare that they have no competing interests.

## Pre-publication history

The pre-publication history for this paper can be accessed here:


